# Antibiotic treatment increases yellowness of carotenoid feather coloration in male greenfinches (*Chloris chloris*)

**DOI:** 10.1038/s41598-021-92598-x

**Published:** 2021-06-24

**Authors:** Mari-Ann Lind, Tuul Sepp, Kristiina Štšeglova, Peeter Hõrak

**Affiliations:** Institute of Ecology and Earth Sciences, University of Tartu, Vanemuise 46, 51014 Tartu, Estonia

**Keywords:** Ecophysiology, Animal physiology

## Abstract

Carotenoid plumage coloration is an important sexually selected trait in many bird species. However, the mechanisms ensuring the honesty of signals based on carotenoid pigments remain unclear. It has recently been suggested that intestinal integrity, which is affected by gut parasites and microbiota and influences nutrient absorption and acquisition, mediates the relationship between carotenoid ornamentation and individual quality. Here, we test whether carotenoid plumage coloration in greenfinches (*Chloris chloris*) is affected by the treatment of an antibiotic or an antiparasitic drug. We captured wild greenfinches (N = 71) and administered anticoccidial medication toltrazuril (TOLTRA) to one group, antibiotic metronidazole (METRO) to the second group to target trichomonosis, and the third group received no medication. In the METRO group, feathers grown during the experiment had significantly higher chroma of yellow parts, but there was no effect of TOLTRA on feather chroma. The results suggest that METRO increased the efficiency of carotenoid modification or deposition to the feathers rather than nutrient acquisition and/or freed energy resources that could be invested in coloration. Alternatively, though not measured, METRO might have affected microbial community and host physiology as microbial metabolites can modulate mitochondrial and immune function.

## Introduction

Carotenoid ornaments are important sexual signals that play a crucial role in mate choice in many vertebrate species, including birds. Females often prefer males with brighter and more intense carotenoid feather coloration, and accordingly, these males are more likely to attain a mate and invest more in taking care of nestlings^1^. In some species, more colourful males live longer and have higher lifetime reproductive success^2^. The honesty of sexually selected signals must be ensured in order for them to contain valuable information and avoid cheating^3^. However, the mechanisms that guarantee the honesty of carotenoid ornaments are still not clear.

Over decades of research on carotenoid feather coloration, several non-mutually exclusive mechanisms have been suggested. For example, feather coloration has been associated with oxidative stress and antioxidant capacity^4–6^. However, multiple studies have failed to replicate these results^7–9^, and meta-analyses have found only weak relationships between these variables and carotenoid coloration^10^. It has also been proposed that the conversion of carotenoids is sensitive to the functionality of mitochondria, as the enzyme responsible has been suggested to reside within the mitochondrial membrane^11^. This is one example of the “shared pathway hypothesis”, according to which signal honesty could also be maintained when production of the signal is tied to the functionality of vital cellular proceses^12^. In other studies, carotenoids have been associated with resistance to parasites and parasite load^13,14^. A recent meta-analysis found that metabolically converted carotenoids were related to parasite resistance and reproductive and parental quality, but unconverted carotenoids were not^15^.

Parasites can affect carotenoid coloration through the compromised absorption of carotenoids in the gut. Many gut parasites cause inflammation of the gut surface that can reduce the absorption of carotenoids and overall energy acquired from the food^16^. Among birds, protozoan coccidian parasites are widely distributed gut parasites that have been shown to affect digestive efficiency. Coccidia are intracellular parasites, and their asexual and sexual multiplication inside of the intestine's epithelial cells damages the animal’s ability to acquire nutrients from food^17,18^. In poultry, coccidian parasites cause extensive damage to the intestinal villi^19^, inflammation of the gut surface^20^, leaky gut^17^, and reduced digestive efficiency^18,21^. The damage caused by coccidiosis to the intestines has been associated with malabsorption of carotenoids^16,22,23^ and other nutrients^17,18^. In captive greenfinches (*Chloris chloris*), goldfinches (*Carduelis carduelis*), and Nashville warblers (*Vermivora ruficapilla*), Isosporan coccidia caused thickening of the intestinal wall^24,25^, which in turn correlated negatively with weight^25^. Coccidia have been shown to affect carotenoid coloration in wild birds^14,26^. However, it is unclear if these effects occur through general condition decline or specifically impact gut integrity.

Other parasites, such as *Trichomonas gallinae* and pathogenic bacteria, may not directly affect intestinal integrity, but decrease the amount of energy available to the individual from food. Protozoan *T. gallinae* is a parasite that causes lesions in the upper digestive tract in several species of birds, which in severe cases can block the esophagus and make the passage of food impossible^27^. Avian trichomonosis caused by *T. gallinae* is historically common in pigeons and raptors. However, since 2005, it has been reported as an emerging disease in greenfinches in Europe, causing epidemics that have resulted in high mortality and a decline in populations of wild greenfinches^28,29^. Avian trichomonosis is an example of a relatively recent phenomenon in which a parasite spills over to another taxonomic group with drastic effects on a new host species.

Due to their well-established effect on growth rate and feed conversion, antibiotics are often administered prophylactically in poultry^30,31^. It is not entirely clear how antibiotic growth promoters elicit its beneficial effects, but it has been shown that they do not exert growth-promoting properties in germ-free animals. Thus, the antimicrobial effect is most likely the key factor in enhancing growth performance. Suggested mechanisms of antibiotic growth promoters include reducing the severity of subclinical infections, reducing the microbial use of nutrients, and improving absorption of nutrients. Antibiotic growth promoters in broiler feed has reported to cause shifts in the gut microbiota composition. These shifts can affect the morphology of the gut wall, modulate immune reactions, and impact the host’s energy expenditure (reviewed in^30^). For example, antibiotic supplementation (methylene disalicylate and virginiamycin) with feed increased the body weight of broiler chickens compared to controls at several time points until seven weeks of age and resulted in morphological changes to the intestines of birds^32^. In chickens coinfected with *Eimeria* and *Clostridium*, antibiotic supplementation (methylene disalicylate and virginiamycin) resulted in deeper intestinal crypts, decreased intestinal lesion, and greater weight gain than in unsupplemented control 22 days post-hatch^33^. Following the European Commission’s ban on the use of antibiotics as growth promoters (January 1st, 2006), an increase of some health conditions in poultry has been described, such as necrotic enteritis and dysbiosis^30^.

Although several studies in poultry have detected the effect of coccidian parasites on decreased nutrient absorption and carotenoid levels, few studies have considered this mechanism in the coloration of wild birds. In contrast to poultry, where the most economically relevant coccidian parasites are from the genus *Eimeria*, the predominant coccidian genus in many wild species is *Isospora*, which might have different effects on the host. However, some studies in wild birds have revealed similar results to poultry. Experimental infection of greenfinches with coccidian parasites showed parallel patterns of decreased carotenoid, serum albumin, and triglycerides in serum and decreased carotenoids in the feathers of greenfinches, which points to reduced digestive and absorption capacity^14^. Infection with *Isospora* sp. coccidians increased fat content in the faeces of greenfinches^34^. Carotenoids are fat-soluble, thus carotenoid deficiency and malabsorption of lipids are likely related^35^. In house finch (*Haemorhous mexicanus*) redness of carotenoid coloration correlated with digestive efficiency —individuals with redder ornaments absorbed more fats from their diet^36^.

The effect of other infections, such as trichomonosis, on carotenoids acquired through diet, has not received much attention. However, in greenfinches, it has been shown that melanin, but not carotenoid coloration, predicted mortality, and black parts of tail feathers became darker in generations emerged subsequent to the outbreak of trichomonosis epidemic, while no change was observed in the yellow parts of feathers^37^. Therefore, different parasites might induce selection pressure on different components of bird coloration. Apart from the growth rate in poultry, not much is known about how the microbiome affects the production of condition-dependent traits. Antibiotics have a well-known effect on altering the composition of the gut microbiome. However, to our knowledge, the impact of experimental administration of antibiotics on sexually selected traits has not been tested previously in wild birds.

This study aimed to test whether treatment with two different antimicrobial medications affected plasma carotenoid levels and carotenoid-based feather coloration in greenfinches. The chroma of the yellow parts of feathers is related to carotenoid content in the feathers of greenfinches. Greenfinches in our study population are naturally infected by coccidian parasites (*Isospora* spp.)^39^, and we previously detected characteristic symptoms of trichomonosis in some birds that have died in the aviary^40^. We treated the birds with the anticoccidial drug Toltrazuril (TOLTRA), which is designed specifically to treat coccidiosis and lacks known effects on microbes other than apicomplexans^41^. Toltrazuril affects the respiratory mechanism of coccidia by interfering with nuclear division and mitochondrial activity^42^. The other group received nitroimidazole group antibiotic, metronidazole (METRO), which targets protozoan parasites of *Trichomonas* genus and a broad spectrum of anaerobic bacteria, e.g. gram-negative bacteria genera *Bacteroides*, *Helicobacter*, *Prevotella*, and gram-positive bacteria such as genus *Clostridium*^27^. METRO converts into nitroso free radicals in the cytoplasm of bacteria or in specific organelles in the protozoa, which inhibits DNA synthesis and damages DNA by oxidation. The drug is only active against bacteria with anaerobic metabolisms and some microaerophils, as aerobic cells lack electron-transport proteins with sufficient negative redox potential^43^.

As carotenoids have been associated with signalling overall parasite resistance, we hypothesized that medicating birds with either TOLTRA or METRO would result in higher plasma carotenoids and higher chroma in lab-grown feathers compared to the control group. Our study design allowed us to distinguish between the effects of treatment against Isospora coccidian parasites (affecting intestinal integrity) vs. treatment against *Trichomonas* parasites (affect nutrient availability but not nutrient absorption in the gut) and various anaerobic bacteria (might affect general health state). Accordingly, we predicted that if intestinal tract integrity is the key factor linking intestinal parasite infection to carotenoid coloration, treatment with either of the drugs (TOLTRA or METRO) would affect plasma carotenoid concentration. Alternatively, if the key factor is overall health state, then birds treated with METRO would show a greater increase in plumage carotenoid coloration. It should be noted however that in our study, we did not directly measure the changes in the microbial community or gut integrity, so our set-up can provide only indirect evidence to support these hypotheses.

## Methods

### Study system and ethics statement

The study was conducted under license from the Estonian Ministry of the Environment (Licence # 1–4.1/11/100, issued on 23rd March 2011), and the experiment was approved by the Committee of Animal Experiments at the Estonian Ministry of Agriculture (decision # 95, issued on 17th January 2012). All experiments were performed in accordance with relevant guidelines and regulations. The study was carried out in compliance with the ARRIVE guidelines. Wild male greenfinches (N = 71) were captured in mist nets at bird feeders in a garden in the city of Tartu, Estonia (58°22′N; 26°43′E) on 5th, 6th, and 8th January 2015. Greenfinches are gregarious medium-sized (c. 28 g) seed-eating passerines native to the western Palearctic region^44^. Males are more colourful with yellow, carotenoid-based^45^ markings on the sides of the tail feathers, primaries, primary coverts, and breast, while females lack full yellow tints in their plumage^44^. Greenfinches incorporate two main carotenoids − canary xanthophylls A and B − into feathers to develop the yellow colour. Canary xanthophylls A and B are metabolically converted from dietary lutein and zeaxanthin^46,47^. The birds were housed indoors in individual cages (27 × 51 × 55 cm) with sand-covered floors in a single room where they had visual contact with their neighbours. The average temperature in the aviary during the experiment was 15.5 ± 1.0° (SD) °C, and average humidity was 57 ± 7 (SD) %. The birds were supplied ad libitum with sunflower seeds and tap water, and were exposed to a natural day-length cycle using artificial lighting by luminophore tubes. The birds were released back to their natural habitat on 3rd March 2015.

**Figure 1:**
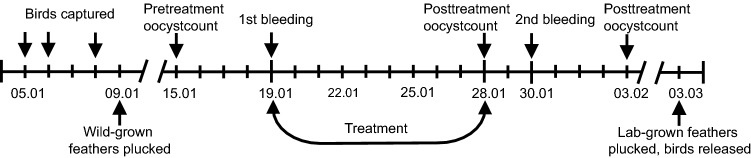
Timeline of the experiment. The dates are in DD/MM format.

We divided birds into three approximately equal-sized groups. The groups did not differ in age composition (approximately an equal number of both, yearlings vs. older birds, in every group, determined based on plumage characteristics), body mass (recorded on 19th January, F_2,68_ = 0.20, p = 0.82), and coccidian infection intensity (recorded on 15th January, F_2,69_ = 0.045, p = 0.96). The timeline of the study is depicted in Fig. 1. On the evening of 19th January, the birds in the two groups subjected to the medication treatment started to receive either TOLTRA (24 birds) or METRO (24 birds) with their carotenoid-enriched drinking water. Twenty-three control birds received just carotenoid-enriched water. Birds in the anticoccidial medication group received a two ml/L solution of Intracox Oral (Interchemie, Castenary, the Netherlands), containing 25 mg/L toltrazuril. METRO (Fresenius Kabi Polska, Kutno, Poland) was administered at a concentration of 400 mg/L. Both drugs were dissolved in carotenoid solution (1 ml/L mix of lutein and zeaxanthin (20:1, w/w), prepared from OroGlo brand 15 Liquid Pigmenter with 15 g/kg xanthophyll activity (Kemin AgriFoods Europe, Herentals, Belgium). The concentration of carotenoid supplement in drinking water was chosen based on previous experiments with greenfinches^48^. Carotenoids were added to the drinking water to compensate for the naturally low carotenoid content of sunflower seeds. Medication lasted for ten days, and carotenoid supplementation lasted until the birds were released. We took blood samples from the birds on 19th January and 30th January to record the effects of treatments on plasma carotenoid concentrations. We weighed the birds after trapping, on 19th January, on 30th January, and before the release on 3rd March. Blood sampling of birds took place in the mornings before the lights turned on. We collected the samples for assessment of coccidian infection intensity on 15th January, on 28th January, and 3rd March. The study duration (9th January–3rd March) was based on the time it takes greenfinches to regrow plucked feathers^49^.

### Measurement of chroma

**Figure 2:**
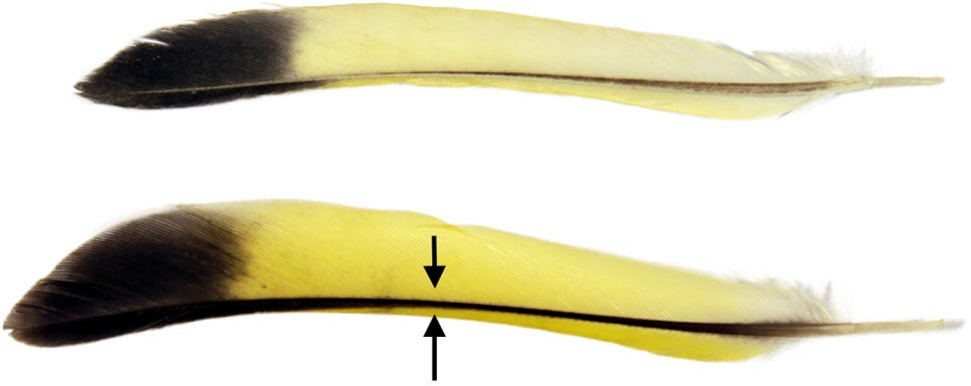
Measurement of feather chroma. The arrows indicate positions where chroma of yellow feather coloration was measured. Upper – lab-grown feather, lower – wild-grown feather.

We plucked the left and right outermost wild-grown tail feathers (rectrices) on 9th January from 71 birds. Replacement feathers grown during the study (lab-grown feathers) were collected on 3rd March, before the release of the birds. We placed collected feathers into a plastic bag and stored them in the dark until measurements were carried out. Locations of colour measurements on feathers are indicated in Fig. 2. The colour was measured from the feathers placed on a black background, in an area of approximately one mm^2^, of the visible surface of the feather, using a spectrophotometer (Ocean Optics S2000) as described by L. Saks and co-authors^38^. To estimate colour, we calculated values of chroma^50^. Chroma can be understood as a measure of the ‘purity’ or ‘saturation’ of colour and has been shown to correlate with the actual carotenoid concentration of feathers. Details for calculations of chroma are described by L. Saks and co-authors^38^. The chroma measurements of left and right feathers were repeatable in lab-grown feathers and wild-grown feathers (r = 0.7, F_60;61_ = 5.1, p < 0.0001)^51^.

### Infection intensity and blood analyses

We collected faecal samples to determine coccidian (*Isospora* spp.) infection intensity from all the birds on 15th January, 28th January, and 3rd February, as described by R. Meitern and co-authors^34^, and all birds appeared naturally infected. We have previously shown that repeatability of infection intensity, measured from two faecal samples collected at the same time, was high (0.91, F = 20.34, p < 0.0001, n = 20)^52^. Diagnosis of trichomonosis requires either sacrificing the birds or PCR-based techniques, which were not available to us during the study^27^. Therefore, we were not able to collect data on the infection status of trichomonads for this study, but previous investigations of dead birds have confirmed that trichomonosis is present in the Estonian greenfinch population. For instance, three birds of the five that died in captivity in our lab during 2013 showed apparent symptoms of trichomonosis but the prevalence of sublethal levels of infection is not known^40^. The concentration of carotenoids was determined spectrophotometrically from 15 μl of plasma, diluted in acetone as described elsewhere^7^. We assessed the nutritional state based on plasma triglycerides, as described by R. Meitern and co-authors^34^. Plasma triglycerides increase during the transport of fat to the adipose tissue and energy-consuming organs and indicate the amount of lipids absorbed and changes in fat stores^53^.

### Statistics

We analysed the effect of experimental manipulation on feather chroma with one-way ANOVA. The assumptions of parametric tests were met (normality of residuals, homogeneity of variances). We used Tukey’s post hoc test to determine the differences between the groups. The effect of experimental manipulation on coccidian infection intensity, plasma carotenoids, plasma triglycerides, and body mass were examined with repeated measures ANOVA, assuming that the effect of treatment would be revealed by a significant ‘time × treatment’ interaction term. All tests were two-tailed, with a P-level below 0.05 as a criterion for significance. Sample sizes varied between analyses due to our inability to collect sufficient good-quality blood samples from all birds. Analyses were performed with Statistica v. 12 (Statsoft Inc., Tulsa, OK). We only examined male greenfinches as carotenoid-based signal traits are more pronounced in males in this species, and the addition of another factor (sex) would have decreased test power. Female birds were used in a different study^34^. We also explored the correlation between chroma, infection intensity, body mass, plasma carotenoids, and plasma triglycerides. All data generated or analysed during this study are included in Supplementary Data.

## Results

### Treatment’s effect on feather chroma

**Figure 3:**
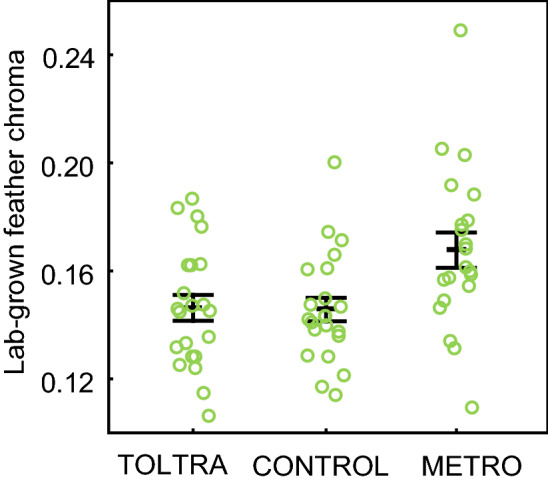
The effect of treatment on feather chroma. The treatment affected chroma of the yellow parts of the lab-grown feathers—birds that received metronidazole (METRO) medication had higher chroma than in the control group and in the toltrazuril (TOLTRA) group. Whiskers denote mean ± s.e.m.

**Figure 4:**
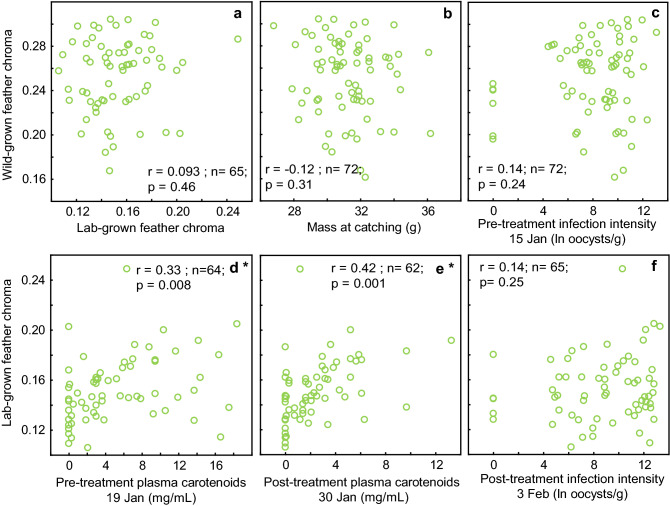
Correlations between feather chroma and physiological and phenotypical traits. Asterisks after the panel letter indicate significant relationships.

The experimental treatment significantly affected the chroma of the yellow parts of the lab-grown tail feathers (ANOVA F_2,62_ = 5.6, p = 0.006, Fig. 3). Birds that received METRO had higher chroma than those in the control group (Tukey’s post hoc, p = 0.012) and in the TOLTRA group (Tukey’s post hoc p = 0.016), whereas there was no difference in the chroma between birds treated with TOLTRA and the control group. There was no difference in the chroma in the wild-grown feathers between the experimental groups (F_2,68_ = 0.43, p = 0.70) and no correlation between chroma of wild-grown and lab-grown feathers (r = 0.093, n = 65, p = 0.46; Fig. 4). Nor were there any relationships between body mass and feather chroma at any point measured (Fig. 4; Supplementary Table S1). Age of birds (juvenile vs. adult) did not affect chroma in wild-grown feathers (ANOVA F_1, 68_ = 1.31, p = 0.26) and lab-grown feathers (ANOVA F_1, 63_ = 0.073, p = 0.79).

### Infection intensity, plasma biomarkers, and body mass

**Figure 5:**
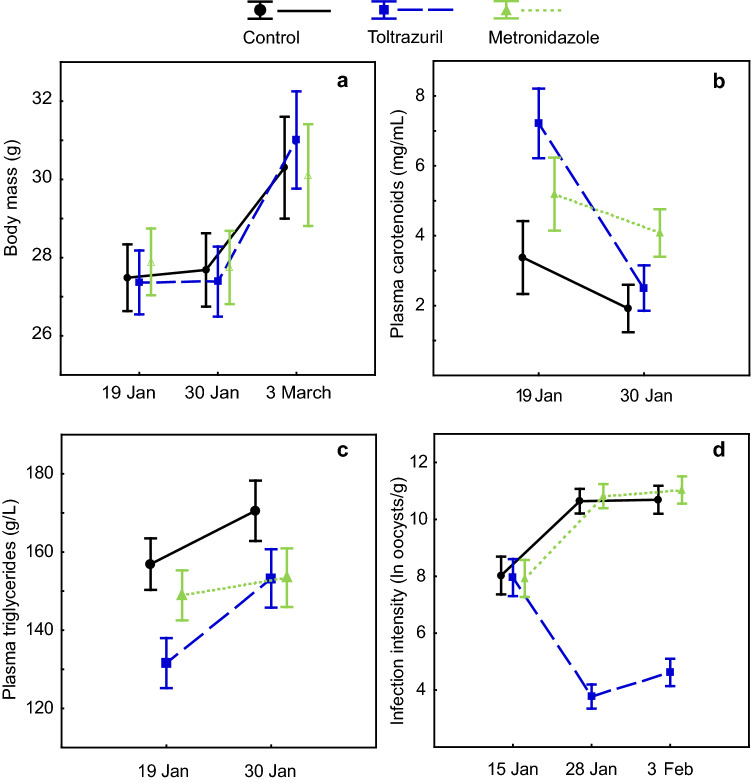
The effect of treatment on body mass, plasma carotenoids, plasma triglycerides, and coccidian infection intensity. (**a**) Treatment did not affect the body mass of birds. (**b**) Treatment had an effect on plasma carotenoids—carotenoids were significantly higher in the toltrazuril group pre-treatment, but after medication, there were no differences between the groups. (**c**) Treatment did not affect plasma triglycerides. (**d**) Medication with TOLTRA significantly reduced the intensity of coccidian infection, while birds medicated with metronidazole (METRO) did not differ from the control group (modification of a previously published figure by R. Meitern and co-authors^34^ (https://doi.org/10.1002/ece3.2575, licensed under CC BY 4.0 https://creativecommons.org/licenses/by/4.0/). The effects were examined with repeated measures ANOVA (Table 1). Whiskers denote mean ± s.e.m.

**Figure 6:**
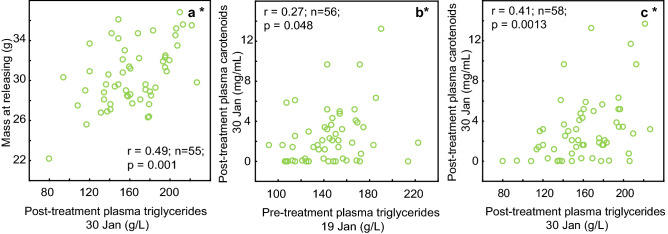
Correlations between physiological and biochemical traits. Asterisks after the panel letter indicate significant relationships.

Treatment did not affect the body mass of birds (Table 1, Fig. 5). There were correlations between body mass and triglycerides on 19th January, on 30th January (Supplementary Table S1), and between body mass at releasing the birds on 3rd March (r = 0.49, n = 55, p = 0.001; Fig. 6).

**Table 1:** Effects of treatment on infection intensity, plasma carotenoid, triglycerides and body mass. See Fig. 5 for the directions of the effects. Effects on infection intensity, body mass, and plasma triglycerides have been previously reported by R. Meitern and co-authors in^34^ (https://doi.org/10.1002/ece3.2575, licensed under CC BY 4.0 https://creativecommons.org/licenses/by/4.0/).

	Effect	F_df_	p
Infection intensity	Treatment	43.9 _2,65_	** < 0.00001**
	Time	2.5 _2,130_	0.087
	Time*treatment	24.5 _4,130_	** <0.00001**
Plasma carotenoids	Treatment	2.67 _2,62_	0.078
	Time	21.82 _1,62_	**0.00002**
	Time*treatment	5.02 _2,62_	**0.0095**
Triglycerides	Treatment	2.94 _2,47_	0.063
	Time	12.04 _1,47_	**0.0011**
	Time*treatment	1.71 _2,47_	0.19
Body mass	Treatment	0.01 _2,65_	0.99
	Time	78.11 _2,124_	** <0.00001**
	Time*treatment	1.58 _4,124_	0.18

The effects of treatment on coccidian infection intensity have been reported in a previously published article on this study by R. Meitern and co-authors^34^. Medication with TOLTRA significantly reduced the intensity of coccidian infection while METRO had no effect and did not differ from the control group (Table 1, Fig. 5 and previously reported in^34^). Pre-treatment infection intensity (ln-transformed oocysts count) did not correlate with chroma of wild-grown feathers (r = 0.14, n = 72, p = 0.24; Fig. 4) and post-treatment infection intensity (measured on 3rd February) did not correlate to chroma of lab-grown feathers (r = 0.14, n = 65, p = 0.25, Fig. 4). Neither were there any other relationships between infection intensity and wild-grown feather chroma or lab-grown feather chroma at any dates measured (Supplementary Table S1).

The treatment had an effect on plasma carotenoids (Table 1, Fig. 5). Plasma carotenoids declined in all groups, but the decline was steepest in the TOLTRA group. Carotenoids were significantly higher in the TOLTRA group pre-treatment (ANOVA F_2,67_ = 3.73, p = 0.03), but after medication, there were no differences between groups (ANOVA F_2,63_ = 2.66, p = 0.08). Effects of treatment on plasma triglycerides were previously reported by R. Meitern and co-authors^34^. No significant effect of treatment on plasma triglycerides was detected (Table 1, Fig. 5). Plasma triglycerides on 19th and 30th January correlated positively with plasma carotenoids measured on 30th January (r = 0.24, n = 56, p = 0.048; r = 0.41, n = 58 p = 0.0013¸ Fig. 6). We also detected that chroma of lab-grown feathers and plasma carotenoids on 19th January and 30th January were correlated (r = 0.33, n = 64, p = 0.008; r = 0.42, N = 62, p = 0.0006; Fig. 4). However, there were no correlations between plasma carotenoids and chroma of wild-grown feathers on neither of the days (Supplementary Table S1).

## Discussion

Carotenoid coloration is suggested to signal both parasite load and resistance of the individual. Our goal was to test whether parasites affect yellow feather coloration through decreased nutrient acquisition. We administered anticoccidial drug TOLTRA or antibiotic METRO, which targets *Trichomonas* protozoa and anaerobic bacteria, to wild-caught captive greenfinches. We found that the treatment with METRO resulted in significantly higher chroma of yellow parts of the feathers, whereas feather colour in birds who received anticoccidial TOLTRA did not differ from the control. There were no significant differences in plasma carotenoids nor in plasma triglycerides between the groups after the treatment, indicating that treatment eliminated prior differences in absorption or availability of these nutrients. Coccidian infection intensity significantly declined in the TOLTRA group, but there were no differences between the METRO and the control group. Treatment did not affect body mass.

We suggest that treatment with antibiotic METRO attenuated the negative effects of trichomonosis, or alternatively, the potential unknown co-infection between several pathogenic bacteria, or between trichomonas and pathogenic bacteria, which freed additional energetic resources that could be invested in ornamentation^13^. One might speculate that antibiotic METRO might have induced change in gut microbiota composition. Some metabolites of microbiota, such as short-chain fatty acids (SCFAs), can affect mitochondrial function^54,55^ resulting in the improvement of vital cellular processes that also influence modification and deposition of carotenoids to the feathers^12^. There was no effect of treatment on yellow feather coloration in the TOLTRA group. One possible explanation could be that birds might have been naturally infected with mildly virulent strains of coccidia that had little effect on their health before the treatment. For instance, it has been demonstrated previously in captive greenfinches that coccidian strains infecting different individuals vary largely in their virulence and hosts differ in resistance^52^.

One explanation for why birds who received METRO grew more saturated yellow feathers might be that METRO had an effect on the co-infection dynamics that freed additional energy resources which could be allocated to production of ornaments. Most individuals in the wild are faced with co-infection of several parasites or several different strains of one parasite^56^, and indeed, all the greenfinches in our study were naturally infected with *Isospora* parasites. We suggest that the wide spectrum antibiotic METRO acted on *Trichomonas gallinae* and on some other (bacterial) infections unknown to us. Although we were not able to diagnose trichomonosis in birds used in this study, previous investigations of dead birds have confirmed that trichomonosis is present in Estonian greenfinch population^40^. The prevalence of Trichomonas parasites has been measured in the greenfinch population in Hesse, Germany, where it showed a high prevalence with 25% of the population being infected and the highly pathogenic strain was widespread^57^. Co-infection can affect the virulence of the parasites^58^, but also the host’s immune responses^56^. Israeli sparrows (*Passer domesticus biblicus*) that were simultaneously infected by Isospora and *Leucocytozoon gentili* experienced more severe symptoms of coccidiosis^59^. Carotenoid feather coloration was paler in blue tits (*Cyanistes caeruleus*) who were infected by different numbers of blood parasite genera compared to those infected by just one^13^. Necrotic enteritis in poultry has been shown to occur when birds with pre-existing mild or asymptomatic coccidiosis are infected with pathogenic *Clostridium* spp. strains (reviewed by^60,61^). Members of *Clostridium* genus are sensitive to METRO, as are many other anaerobic bacteria^62^. It is possible that by administering METRO, the birds were relieved from the adverse effects of co-infection between coccidia and pathogenic bacteria. However, we did not measure microbiome and for definitive evidence this should be tested in future studies. In conclusion, allocating energy between fighting multiple infections and other physiological functions can result in duller ornaments because carotenoid uptake, transportation, modification, and deposition to ornaments is energetically demanding in itself^63^. Thus, carotenoids might signal energetic state of the animal.

We suggest that the effect of METRO on yellow feather coloration might have resulted from improved vital cellular processes that are associated with carotenoid modification and carotenoid deposition into feathers^12^. It has been proposed that the modification of carotenoids is related to the capacity to produce energy in the membranes of mitochondria^64^. Antibiotics can affect host energy metabolism through altering gut microbiota composition. In addition to antiparasitic effects, antibiotics can be harmful as they can alter the normal microflora. However, recent research has found that some antibiotics can increase the abundance of beneficial bacteria (reviewed by^65^). These bacteria can modulate the intestinal immune system and are important producers of short-chain fatty acids (SCFAs), which act as regulatory molecules that influence energy homeostasis and mitochondrial function (reviewed by^54,55^). Due to their common ancestry, mitochondria share several structural and functional features with prokaryotes, hence many bacterial metabolites (such as SCFAs, secondary bile acids, lipopolysaccharides, urolithins) have been shown to both affect mitochondria and have other regulatory and immunomodulatory effects (reviewed by^54,55,66^). It is possible that although METRO treatment did not result in higher nutrient absorption, it might have elicited a shift in gut microbiota composition that improved the host’s cellular processes. Our results are consistent with the shared pathway hypothesis; however, future experimental work into connections between mitochondrial function, ornamentation, and microbiota is needed.

Treatment with TOLTRA significantly reduced coccidian infection intensity but did not affect yellow feather-chroma in lab-grown feathers. Coccidian infection intensity did not correlate to either wild-grown or lab-grown feather coloration at any date measured. The lack of any effect from treatment with TOLTRA and coccidian infection intensity on feather colour might have been due to low virulence of natural infection and that the strains of coccidia were familiar to the birds; greenfinches have better tolerance to their ‘own’ previously acquired parasites than to novel strains^52^. Infection intensities were higher when birds were infected with strains that originated from several hosts, opposed to when birds were inoculated with coccidia strains from one single host^52^, which indicates a variation in virulence in *Isospora* coccidia in greenfinches. We suggest that the naturally occurring coccidian infection did not cause considerable intestinal damage in our study system that would have had any effect on the absorption of carotenoids. It has been suggested that birds in the wild can tolerate *Isosporan* parasites well but can experience more serious illness under stressful conditions (e.g. captivity), poor sanitary conditions, or with concurrent infections^24,59^. Greenfinches, however, have previously been shown to tolerate captivity well based on low stress hormone levels in captivity^67^ and several haematological indices^68^. Thus, the stress of captivity might not have been severe enough to induce exacerbation of the coccidian infection.

The effect of treatment on plasma carotenoids and triglycerides suggests that there were no differences in carotenoid and triglyceride absorption between groups after the treatment. Plasma carotenoid levels were significantly higher before the treatment in birds who subsequently received TOLTRA. Plasma characteristics from blood were measured after the experiment was carried out and could not be considered while grouping the birds; thus, pre-treatment carotenoids in the TOLTRA group are higher by chance. After the treatment plasma carotenoids did not differ between groups; however, plasma carotenoid levels declined in all groups. We suggest that carotenoid levels in all the birds fell to approximately the same levels in the lab conditions because all birds received a constant amount of carotenoids. Negative effect of TOLTRA on plasma carotenoid levels cannot be ruled out, however, we are not aware of any such findings. It is thus more likely that equal carotenoid availability in the diet during captivity was responsible for uniform plasma carotenoid levels in all our treatment groups. Similar decrease in plasma carotenoid levels during captivity has been recorded in our previous experiments with greenfinches^7,14,68^. Across all treatment groups, plasma carotenoids on both blood-sampling dates were positively correlated with yellow feather chroma in lab-grown feathers (but not with wild-grown feathers). However, similar post-treatment carotenoid plasma levels support our suggestion that more saturated yellow lab-grown feathers in the METRO group were not only related to the plasma carotenoid levels, but also might have involved a component of increased efficiency in carotenoid modification or deposition.

Opposed to the effects of antibiotics in poultry, treatment did not affect the body mass in greenfinches. In poultry studies, improved body mass gain with antibiotic growth promoters is usually observed during the period between post-hatch and maturation. The birds in our study were either adults or yearlings who had reached their adult weight by the time of the study. Therefore, in our study system, the effect of treatment on feather coloration was independent of body mass and the body weight of birds might have depended more on other factors rather than mild to moderate infection. We did not find significant effect of treatment on plasma triglycerides. However, there was positive correlation between triglycerides and body mass on 19th January, 30th January and 3rd March. Carotenoids on 30th January (but not on 19th January) positively correlated with both measurements on triglycerides. Although there are relations between body weight and triglycerides, as well as between triglycerides and plasma carotenoids, these do not reflect directly in the carotenoid coloration, based on the results of this study.

To conclude, treatment of captive greenfinches with two antimicrobial agents – narrow-spectrum anticoccidial Toltrazuril and a wide spectrum antibacterial/anti-trichomonad Metronidazole did not affect plasma carotenoid levels, indicating that neither of our treatments affected carotenoid absorption. Among the birds treated with metronidazole, feathers grown during the experiment had significantly higher yellow chroma, which is indicative of higher deposition of dietary carotenoids into feathers. The latter finding provides indirect support to the hypothesis that the microbiome is important for carotenoid metabolism, transportation, and/or deposition. Assuming that microbial metabolites can modulate mitochondrial and immune function by altering the efficiency of vital cellular processes, our findings are also consistent with the shared pathway hypothesis whereby the mechanisms of production of ornaments share functional pathways with core-life supporting pathways. Specific mechanisms into how the microbiome relates to carotenoid metabolism and signalling thus await further investigation.

## Supplementary Information

Supplementary Information 1.

Supplementary Information 2.

## Data Availability

All data generated or analysed during this study are included in Supplementary Information.

## References

[1] Hill GE (1991). Plumage coloration is a sexually selected indicator of male quality. Nature.

[2] Cantarero A, Pérez-Rodríguez L, Romero-Haro AÁ, Chastel O, Alonso-Alvarez C (2019). Carotenoid-based coloration predicts both longevity and lifetime fecundity in male birds, but testosterone disrupts signal reliability. PLoS ONE.

[3] Zahavi A (1975). Mate selection—A selection for a handicap. J. Theor. Biol..

[4] Alonso-Alvarez, C. & Galván, I. Free radical exposure creates paler carotenoid-based ornaments: A possible interaction in the expression of black and red traits. *PLoS ONE* **6** (2011). 10.1371/journal.pone.0019403 PMC3083443 21556328

[5] Schantz TV, Bensch S, Grahn M, Hasselquist D, Wittzell H (1999). Good genes, oxidative stress and condition–dependent sexual signals. Proc. R. Soc. Lond. Ser. B: Biol. Sci..

[6] Tomášek O (2016). Opposing effects of oxidative challenge and carotenoids on antioxidant status and condition-dependent sexual signalling. Sci. Rep..

[7] Sild E, Sepp T, Männiste M, Hõrak P (2011). Carotenoid intake does not affect immune-stimulated oxidative burst in greenfinches. J. Exp. Biol..

[8] Mohr AE, Girard M, Rowe M, McGraw KJ, Sweazea KL (2019). Varied effects of dietary carotenoid supplementation on oxidative damage in tissues of two waterfowl species. Comp. Biochem. Physiol. B: Biochem. Mol. Biol..

[9] Costantini D, Møller A (2008). Carotenoids are minor antioxidants for birds. Funct. Ecol..

[10] Simons MJP, Cohen AA, Verhulst S (2012). What does carotenoid-dependent coloration tell? Plasma carotenoid level signals immunocompetence and oxidative stress state in birds—A meta-analysis. PLoS ONE.

[11] Hill GE (2019). Plumage redness signals mitochondrial function in the house finch. Proc. R. Soc. B.

[12] Hill GE (2011). Condition-dependent traits as signals of the functionality of vital cellular processes. Ecol. Lett..

[13] del Cerro S (2010). Carotenoid-based plumage colouration is associated with blood parasite richness and stress protein levels in blue tits (*Cyanistes caeruleus*). Oecologia.

[14] Hõrak P (2004). How coccidian parasites affect health and appearance of greenfinches. J. Anim. Ecol..

[15] Weaver RJ, Santos ES, Tucker AM, Wilson AE, Hill GE (2018). Carotenoid metabolism strengthens the link between feather coloration and individual quality. Nat. Commun..

[16] Tyczkowski JK, Hamilton PB, Ruff MD (1991). Altered metabolism of carotenoids during pale-bird syndrome in chickens infected with *Eimeria acervulina*. Poult. Sci..

[17] Joyner L (1975). Amino-acid malabsorption and intestinal leakage of plasma-proteins in young chicks infected with *Eimeria acervulina*. Avian Pathol..

[18] Sharma V, Fernando M (1975). Effect of *Eimeria acervulina* infection on nutrient retention with special reference to fat malabsorption in chickens. Can. J. Comp. Med..

[19] Pout DD (1967). Villous atrophy and coccidiosis. Nature.

[20] Sanches AWD (2020). Basal and infectious enteritis in broilers under the I See inside methodology: A chronological evaluation. Front. Vet. Sci..

[21] Russell J, Ruff M (1978). *Eimeria* spp.: Influence of coccidia on digestion (amylolytic activity) in broiler chickens. Exp. Parasitol..

[22] Kouwenhoven B, van der Horst CJ (1972). Disturbed intestinal absorption of vitamin A and carotenes and the effect of a low pH during *Eimeria acervulina* infection in the domestic fowl (*Gallus domesticus*). Z. Parasitenkd..

[23] Ruff MD, Fuller HL (1975). Some mechanisms of reduction of carotenoid levels in chickens infected with *Eimeria acervulina* or *E. tenella*. J. Nutr..

[24] Swayne DE, Getzy D, Slemons RD, Bocetti C, Kramer L (1991). Coccidiosis as a cause of transmural lymphocytic enteritis and mortality in captive Nashville warblers (*Vermivora ruficapilla*). J. Wildl. Dis..

[25] Gosbell MC, Olaogun OM, Luk K, Noormohammadi AH (2020). Investigation of systemic isosporosis outbreaks in an aviary of greenfinch (*Carduelis chloris*) and goldfinch (*Carduelis carduelis*) and a possible link with local wild sparrows (Passer domesticus). Aust. Vet. J..

[26] Baeta R, Faivre B, Motreuil S, Gaillard M, Moreau J (2008). Carotenoid trade-off between parasitic resistance and sexual display: An experimental study in the blackbird (*Turdus merula*). Proc. R. Soc. B Biol. Sci..

[27] Amin A, Bilic I, Liebhart D, Hess M (2014). Trichomonads in birds—A review. Parasitology.

[28] Robinson, R. A. *et al.* Emerging infectious disease leads to rapid population declines of common British birds. *PLoS ONE* **5** (2010). 10.1371/journal.pone.0012215 PMC2923595 20805869

[29] Chavatte J-M (2019). An outbreak of trichomonosis in European greenfinches Chloris chloris and European goldfinches *Carduelis carduelis* wintering in Northern France. Parasite.

[30] Huyghebaert G, Ducatelle R, Immerseel FV (2011). An update on alternatives to antimicrobial growth promoters for broilers. Vet. J..

[31] Singer RS, Hofacre CL (2006). Potential impacts of antibiotic use in poultry production. Avian Dis..

[32] Miles RD, Butcher GD, Henry PR, Littell RC (2006). Effect of antibiotic growth promoters on broiler performance, intestinal growth parameters, and quantitative morphology1. Poult. Sci..

[33] Oh S, Lillehoj HS, Lee Y, Bravo D, Lillehoj EP (2019). Dietary antibiotic growth promoters down-regulate intestinal inflammatory cytokine expression in chickens challenged with LPS or co-infected with *Eimeria maxima* and *Clostridium perfringens*. Front. Vet. Sci..

[34] Meitern R, Lind MA, Karu U, Hõrak P (2016). Simple and noninvasive method for assessment of digestive efficiency: Validation of fecal steatocrit in greenfinch coccidiosis model. Ecol. Evol..

[35] Surai P, Speake B, Sparks N (2001). Carotenoids in avian nutrition and embryonic development. 1. Absorption, availability and levels in plasma and egg yolk. J. Poultry Sci..

[36] Madonia C, Hutton P, Giraudeau M, Sepp T (2017). Carotenoid coloration is related to fat digestion efficiency in a wild bird. Sci. Nat..

[37] Hõrak P, Männiste M (2016). Viability selection affects black but not yellow plumage colour in greenfinches. Oecologia.

[38] Saks L, McGraw K, Hõrak P (2003). How feather colour reflects its carotenoid content. Funct. Ecol..

[39] Sepp, T. *et al.* Coccidian infection causes oxidative damage in greenfinches. *PLoS ONE* **7** (2012). 10.1371/journal.pone.0036495 PMC3352913 22615772

[40] Männiste M, Hõrak P (2014). Emerging infectious disease selects for darker plumage coloration in greenfinches. Front. Ecol. Evol..

[41] Hackstein JH (1995). Parasitic apicomplexans harbor a chlorophyll a-D1 complex, the potential target for therapeutic triazines. Parasitol. Res..

[42] Krautwald-Junghanns, M.-E., Zebisch, R. & Schmidt, V. Relevance and treatment of coccidiosis in domestic pigeons (Columba livia forma domestica) with particular emphasis on toltrazuril. *Journal of Avian Medicine and Surgery*, 1–5 (2009). 10.1647/2007-049R.1 19530399

[43] Löfmark S, Edlund C, Nord CE (2010). Metronidazole is still the drug of choice for treatment of anaerobic infections. Clin. Infect. Dis..

[44] Cramp, S. & Perrins, C. Handbook of the Birds of the Western Palearctic. *Volume IV. Terns to Woodpeckers (ed. Cramp, S.)*, 353–363 (1994).

[45] Stradi R, Celentano G, Rossi E, Rovati G, Pastore M (1995). Carotenoids in bird plumage—I. The carotenoid pattern in a series of Palearctic Carduelinae. Comp. Biochem. Physiol. Part B: Biochem. Mol. Biol..

[46] Stradi, R. *The colour of flight: carotenoids in bird plumages*. (Solei Gruppo Editoriale Informatico, 1998).

[47] McGraw K, Hill G, Stradi R, Parker R (2002). The effect of dietary carotenoid access on sexual dichromatism and plumage pigment composition in the American goldfinch. Comp. Biochem. Physiol. B: Biochem. Mol. Biol..

[48] Sepp T, Karu U, Sild E, Männiste M, Hõrak P (2011). Effects of carotenoids, immune activation and immune suppression on the intensity of chronic coccidiosis in greenfinches. Exp. Parasitol..

[49] Hõrak P (2013). Dexamethasone inhibits corticosterone deposition in feathers of greenfinches. Gen. Comp. Endocrinol..

[50] Endler JA (1990). On the measurement and classification of colour in studies of animal colour patterns. Biol. J. Lin. Soc..

[51] Lessells C, Boag PT (1987). Unrepeatable repeatabilities: A common mistake. Auk.

[52] Hõrak P, Saks L, Karu U, Ots I (2006). Host resistance and parasite virulence in greenfinch coccidiosis. J. Evol. Biol..

[53] Jenni-Eiermann S, Jenni L (1994). Plasma metabolite levels predict individual body-mass changes in a small long-distance migrant, the Garden Warbler. Auk.

[54] Saint-Georges-Chaumet Y, Edeas M (2015). Microbiota–mitochondria inter-talk: Consequence for microbiota–host interaction. Pathogens Dis..

[55] Franco-Obregón A, Gilbert JA (2017). The microbiome-mitochondrion connection: Common ancestries, common mechanisms, common goals. mSystems.

[56] Paterson S (2013). The immunology and ecology of co-infection. Mol. Ecol..

[57] Quillfeldt P (2018). Prevalence and genotyping of Trichomonas infections in wild birds in central Germany. PLoS ONE.

[58] Kinnula H, Mappes J, Sundberg L-R (2017). Coinfection outcome in an opportunistic pathogen depends on the inter-strain interactions. BMC Evol. Biol..

[59] Gill H, Paperna I (2008). Proliferative visceral Isospora (atoxoplasmosis) with morbid impact on the Israeli sparrow Passer domesticus biblicus Hartert, 1904. Parasitol. Res..

[60] Shojadoost B, Vince AR, Prescott JF (2012). The successful experimental induction of necrotic enteritis in chickens by Clostridium perfringens: A critical review. Vet. Res..

[61] Williams R (2005). Intercurrent coccidiosis and necrotic enteritis of chickens: rational, integrated disease management by maintenance of gut integrity. Avian Pathol..

[62] Freeman CD, Klutman NE, Lamp KC (1997). Metronidazole. Drugs.

[63] Hill GE (2000). Energetic constraints on expression of carotenoid-based plumage coloration. J. Avian Biol..

[64] Hill GE (2014). Cellular respiration: The nexus of stress, condition, and ornamentation. Integr. Comp. Biol..

[65] Ianiro G, Tilg H, Gasbarrini A (2016). Antibiotics as deep modulators of gut microbiota: Between good and evil. Gut.

[66] Heiss CN, Olofsson LE (2018). Gut microbiota-dependent modulation of energy metabolism. J. Innate Immun..

[67] Lind M-A, Hõrak P, Sepp T, Meitern R (2020). Corticosterone levels correlate in wild-grown and lab-grown feathers in greenfinches (*Carduelis chloris*) and predict behaviour and survival in captivity. Horm. Behav..

[68] Sepp T, Sild E, Horak P (2010). Hematological condition indexes in greenfinches: Effects of captivity and diurnal variation. Physiol. Biochem. Zool..

