# Supplementary material for: Antibiotic treatment increases yellowness of carotenoid feather coloration in male greenfinches (Chloris chloris)
Source: Sci Rep. 2021 Jun 24;11:13235. doi: 10.1038/s41598-021-92598-x (PMC8225797; doi:10.1038/s41598-021-92598-x)

**Antibiotic treatment increases yellowness of carotenoid feather coloration in male greenfinches (Chloris chloris)**

**Mari-Ann Lind, Tuul Sepp, Kristiina Štšeglova, Peeter Hõrak**

**Supplementary Table S1. Correlation matrix of the traits measured.** p-values are reported along with test statistics for the ease of assessment of the evidence against the statistical null hypothesis. We did not apply Bonferroni correction as it is too stringent for our set of correlated traits. P-level below 0.05 was considered as a criterion for significance and indicated with red in the table.

Abbreviations: trigl - plasma triglycerides (g/L), Car - plasma carotenoids (mg/mL), mass (g), lncoc - ln-transformed infection intensity (oocysts/g).


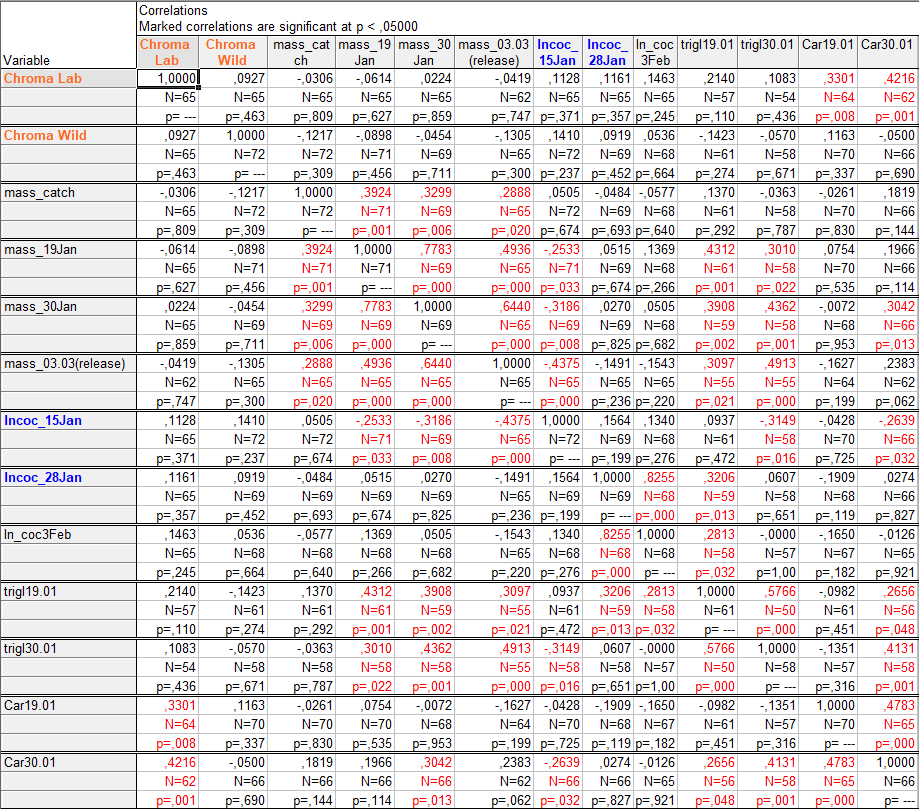

Supplement: Supplementary file 2 — Supplementary Information 2. [file 41598_2021_92598_MOESM2_ESM.docx]
